# Cotton fiber elongation network revealed by expression profiling of longer fiber lines introgressed with different *Gossypium barbadense* chromosome segments

**DOI:** 10.1186/1471-2164-15-838

**Published:** 2014-10-02

**Authors:** Lei Fang, Ruiping Tian, Xinghe Li, Jiedan Chen, Sen Wang, Peng Wang, Tianzhen Zhang

**Affiliations:** National Key Laboratory of Crop Genetics and Germplasm Enhancement, Cotton Hybrid R & D Engineering Center (the Ministry of Education), Nanjing Agricultural University, Nanjing, 210095 China

**Keywords:** CSIL, DEG, Fiber length, Functional enrichment, Metabolic pathway analysis, Transcriptome

## Abstract

**Background:**

Cotton fiber, a highly elongated, thickened single cell of the seed epidermis, is a powerful cell wall research model. Fiber length, largely determined during the elongation stage, is a key property of fiber quality. Several studies using expressed sequence tags and microarray analysis have identified transcripts that accumulate preferentially during fiber elongation. To further show the mechanism of fiber elongation, we used Digital Gene Expression Tag Profiling to compare transcriptome data from longer fiber chromosome introgressed lines (CSILs) containing segments of various *Gossypium barbadense* chromosomes with data from its recurrent parent TM-1 during fiber elongation (from 5 DPA to 20 DPA).

**Results:**

A large number of differentially expressed genes (DEGs) involved in carbohydrate, fatty acid and secondary metabolism, particularly cell wall biosynthesis, were highly upregulated during the fiber elongation stage, as determined by functional enrichment and pathway analysis. Furthermore, DEGs related to hormone responses and transcription factors showed upregulated expression levels in the CSILs. Moreover, metabolic and regulatory network analysis indicated that the same pathways were differentially altered, and distinct pathways exhibited altered gene expression, in the CSILs. Interestingly, mining of upregulated DEGs in the introgressed segments of these CSILs based on D-genome sequence data showed that these lines were enriched in glucuronosyltransferase, inositol-1, 4, 5-trisphosphate 3-kinase and desulfoglucosinolate sulfotransferase activity. These results were similar to the results of transcriptome analysis.

**Conclusions:**

This report provides an integrative network about the molecular mechanisms controlling fiber length, which are mainly tied to carbohydrate metabolism, cell wall biosynthesis, fatty acid metabolism, secondary metabolism, hormone responses and Transcription factors. The results of this study provide new insights into the critical factors associated with cell elongation and will facilitate further research aimed at understanding the mechanisms underlying cotton fiber elongation.

**Electronic supplementary material:**

The online version of this article (doi:10.1186/1471-2164-15-838) contains supplementary material, which is available to authorized users.

## Background

Cotton is a commercial fiber crop, producing the most prevalent natural fibers used by the textile industry. Cotton fibers are single-celled seed trichomes that develop from epidermal cells of the ovule, 30% of which differentiate into spinnable fibers [1, 2]. Cotton fibers have four developmental stages, including initiation (-3 to +3 days post-anthesis; DPA), elongation (3–23 DPA), secondary cell wall synthesis (16–40 DPA) and maturation (40–50 DPA) [3–7].

Cotton fiber is an excellent model for studying the mechanisms of plant cell elongation, with peak rates of expansion of >2 mm/day in *Gossypium hirsutum* during the elongation period [5, 8, 9]. In recent years, cotton functional genomics studies have provided new insights into fiber development, and transcriptome profiling has been employed to analyze the early stages of fiber development and the elongation stage in *G. hirsutum*, *G. barbadense* and *G. arboretum* cotton species [1, 10–12]. Mutant analysis in combination with microarray or next generation sequencing provides a powerful approach for discovering fiber developmental mechanisms by comparing gene expression in mutant vs. wild-type plants [11, 13–15]. Phytohormones such as auxins [13, 16, 17], ethylene [11, 18] and brassinosteroids [18, 19] are involved in fiber development. In addition, carbohydrate and lipid metabolisms play important roles in fiber development by providing the plant with cell wall polysaccharides and fatty acids [13, 20, 21]. Some genes encoding members of the cell wall-loosening expansin family are highly expressed in elongating fiber cells [20] and downregulated in fuzzless-lintless mutants [22]. Several studies have elucidated the role of xyloglucan, pectin and the actin cytoskeleton in cotton fiber elongation [23–26]. Transcription factors such as MYB25 and MYB25-like are also involved in fiber development [27–29].

*G. hirsutum*, which represents over 95% of cultivated cotton worldwide, is characterized by high yield and moderate fiber quality. *G. barbadense*, acultivated extra-long staple tetraploid cotton, is characterized by low yield and increased fiber quality (fineness and strength). Chromosome segment introgression line (CSIL) production is an effective method for combining the high yield of *G. hirsutum* with the superior fiber properties of *G. barbadense*. With the exception of a single, homozygous chromosome segment transferred from a donor parent, the remaining genome of each CSIL is the same as that of the recipient parent [30]. CSILs consist of a battery of near-isogenic lines that have been developed to cover the entire genomes of some crops, including *Lycopersicon esculentum* (tomato), *Oryza sativa* (rice), *Triticum aestivum* (wheat) and *Gossypium* (cotton) [30–34].

In this study, we analyzed the transcriptome profiles of longer fiber CSILs containing inserts of various *G. barbadense* chromosome segments in the background of the standard genetic line *G. hirsutum* cv. TM-1, developed in our laboratory [30], using the Illumina HiSeq 2000 platform. These results were further validated by quantitative real-time PCR, and functional enrichment and metabolic pathway analysis were performed on the DEGs. This study showed a network including carbohydrate-, fatty acid-, secondary metabolism-, hormone- and transcription factor-related genes associated with fiber elongation. The goal of this study was to gain new insights into the molecular mechanisms behind superior quality fiber formation and to identify new candidate genes as potential targets for fiber property improvement.

## Methods

### Plant materials

*G. hirsutum* cv. TM-1, the genetic standard line for upland cotton [35], was obtained from the Southern Plains Agricultural Research Center, USDA-ARS, College Station, Texas, USA. *G. barbadense* cv. Hai7124, extra-long staple cotton, is widely grown in China [30, 36]. The detailed method used to develop the CSILs has been described previously [30]. The introgressed *G. barbadense* chromosomal segments were different in all four lines [37].The samples were collected at 5, 10, 15 and 20 DPA, frozen in liquid nitrogen and stored at -70°C.

### RNA isolation and evaluation

Total RNA was extracted from frozen tissue using an improved CTAB extraction protocol [38]. RNAs were evaluated for quality using RNA Pico Chips in an Agilent 2100 Bioanalyzer (Agilent Technologies, Santa Clara, CA, USA). All RNA samples were quantified, and samples with an RNA Integrity Number (RIN) >8 and 28S/18S rRNA band intensity (2:1) were subjected to further analysis.

### Library construction and sequencing

Digital gene expression (DGE) libraries were constructed using an Illumina Gene Expression Sample Preparation Kit according to the manufacturer’s instructions. A total of 24 libraries derived from immature fibers at 5, 10, 15 and 20 DPA were constructed and sequenced using the Solexa Genome Sequencing Analyzer system provided by BGI (Beijing Genomics Institute at Shenzhen, China), which was described in detail previously [39].

### Data processing, statistical evaluation and selection of differentially expressed genes

Raw data reads were filtered by the Illumina pipeline to produce clean data. All low-quality data, such as short tags (<21 nt) and singletons, were removed. A database of 21-base-long sequences was produced beginning with CATG using 37,505 reference genes from the diploid species *G. raimondii* (http://www.phytozome.net). The remaining high quality sequences were then mapped to this database; only a single mismatch was allowed, and more than one match was excluded. Gene expression levels were the summation of tags aligned to different positions of the same gene. Expression levels were expressed as TPM, transcripts per million. To identify DEGs during fiber elongation, pairs of DEG profiles from different libraries were compared. Four fiber developmental periods for the five CSILs were compared with the same period for TM-1, and 20 comparisons were obtained. P- and Q-values were also calculated for every comparison [40]. DEGs were defined as FDR ≤ 0.001, with an absolute value of |log_2_Ratio| ≥ 1, to judge the significance of differences in transcript abundance.

### Digital tag profiling analysis

Genes expressed in more than half of the libraries were used for PCC analysis, and clustering of log2-transformed TPM values of these genes was performed with the “Self-organizing tree algorithm” (SOTA, Multiple Array Viewer software, MeV 4.9.0; http://www.tm4.org/mev.html) [41]. Clustering of DEGs in CSILs at different developmental stages was performed with Cluster3.0 (http://bonsai.hgc.jp/~mdehoon/software/cluster/software.htm).

Mapman was also used to analyze gene enrichment [42] and metabolic pathways based on the KEGG database [43]. GO enrichment and KEGG (Kyoto Encyclopedia of Genes and Genomes) pathway analysis were performed using BLAST2GO (http://www.blast2go.com/b2ghome).

### Quantitative RT-PCR

Quantitative RT-PCR assays were performed in a 7500 Real-Time PCR system (Applied Biosystems, San Francisco, CA, USA). The reactions were performed in a final volume of 20 μL containing 2 μL of diluted cDNA, 10 μL of 2× SYBR mix (Roche, Basel, Switzerland) and 200 nM of forward and reverse primers. Primer lengths were designed to range from 18 to 24 nt using Beacon Designer 7, and PCR amplicon lengths were designed to range from 100 bp to 150 bp. The thermal cycling conditions were 40 cycles of 95°C for 15 s, 60°C for 30 s and 72°C for 30 s. All reactions were run in triplicate, and the cotton *histone3* gene (ACC NO.AF024716) was used as an internal control for normalization of expression levels (F: 5′-GGTGGTGTGAAGAAGCCTCAT-3′, and R: 5′-AATTTCACGAACAAGCCTCTGGAA-3′). The relative gene expression levels were presented as 2-ΔCT. The Pfaffl method was used to analyze expression data [44].

## Results

### Fiber quality of CSILs and TM-1

In this study, four CSILs with longer fibers than the recurrent parent TM-1, containing inserts of various *G. barbadense* chromosome segment(s), were identified. The average fiber length of CSIL-35431, CSIL-31134, CSIL-31068 and CSIL-31044 was 31.33, 30.63, 31.00 and 29.97 mm, respectively, which was significantly longer than that of TM-1, while CSIL-35368 had a shorter fiber length than TM-1, at 27.67 mm (Table 1). The fiber quality of these CSILs showed significant difference compared to the recurrent parent TM-1 and these CSILs provide good materials for the study of fiber elongation and the functional genetic study of cotton fiber trait.

**Table 1:** **Average fiber quality of five CSILs and TM-1**

CSIL	Chromosome segment	Fiber length (mm)	Fiber strength (cN/tex)	Mrc
CSIL-35431	A8(Chr.8) D10(Chr.20)	31.33 ± 1.95**	35.10 ± 2.12**	4.84 ± 0.64
CSIL-31134	A8(Chr.8) D1(Chr.15)	30.63 ± 1.14**	34.73 ± 2.03**	4.59 ± 1.09
CSIL-31068	A9(Chr.9) D12(Chr.26)	30.00 ± 0.89**	31.17 ± 1.75**	4.80 ± 0.78
CSIL-31044	A3(Chr.3)	29.97 ± 0.88**	30.54 ± 1.82	4.50 ± 0.66
TM-1		28.89 ± 0.53	30.11 ± 0.43	4.81 ± 0.75
CSIL-35368	D11(Chr. 20)	27.67 ± 1.18**	28.71 ± 3.19	4.47 ± 0.45

**CSILs have significant difference with TM-1 at the 1% level.

### Gene expression patterns during cotton fiber elongation

To obtain a global view of transcriptome profiles relevant to cotton fiber elongation, we sequenced 24 libraries of elongating fibers from CSILs and their recurrent parent TM-1. The number of raw tag per library ranged from 7.0 to 8.7 million, and the number of clean tags named distinct sequences ranged from 6.8 to 8.5 million. The distribution of unambiguous clean tag mapping to genes was nearly 50%, and 55–60% of reference genes were mapped with unambiguous tags, showing highly similar tendencies for all libraries (Additional file 1: Table S1).

**Figure 1:**
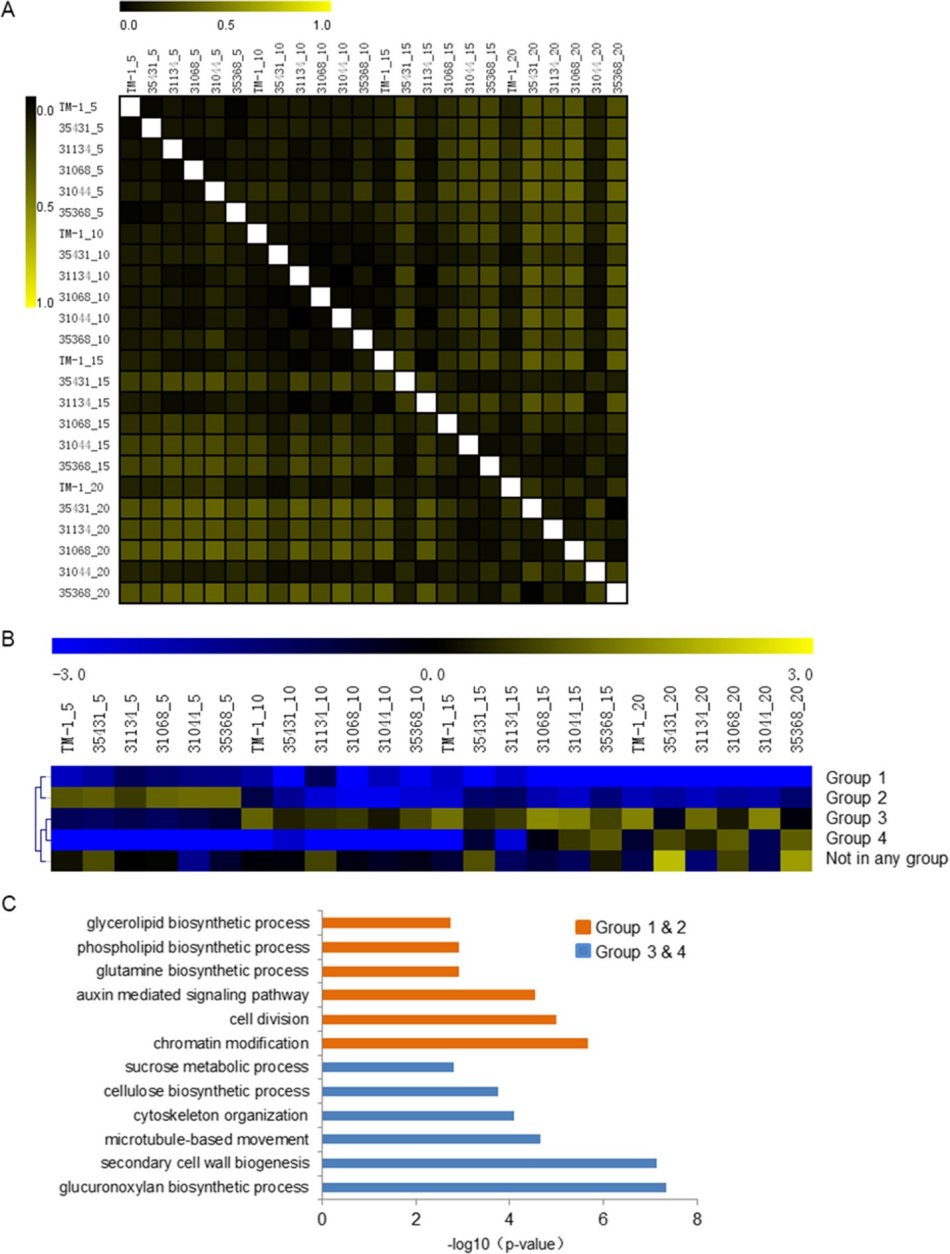
**Statistical analysis of transcript profiling data. (A)** Pearson correlation coefficient analysis of all 24 libraries. **(B)** SOTA clustering analysis of genes in these libraries using Log2 (TPM). **(C)** Distribution of functions of genes in different clusters. Y-axis indicates enriched biological processes. 5, 5 DPA; 10, 10 DPA; 15, 15 DPA; 20, 20 DPA.

A total of 22,153 genes (76.4% of all expressed genes in all libraries) were expressed in more than half of the 24 libraries. To examine the relationship between the experimental samples, Pearson correlation coefficient (PCC) analysis was performed on these genes obtained from all 24 libraries. As shown in Figure 1A, the gene expression profiles in TM-1 showed low similarities at all four stages of fiber elongation, and we also found low similarities in all libraries at the early stages (5 DPA and 10 DPA). However, at later stages (15 DPA and 20 DPA), higher similarities were observed compared to the earlier stages, except for TM-1, which indicates that the gene expression patterns were altered more dramatically in CSILs in the later stages than in the earlier stages, perhaps because the CSILs carried distinct *G. barbadense* chromosomal segments.

To examine gene expression patterns during fiber development, the 22,153 genes were classified into four groups, along with an unclassified group (Figure 1B). Genes in groups 1 and 2 were more highly expressed in the early stage than in the later stage, but genes in groups 3 and 4 showed an opposite expression pattern.

Classification of gene functions showed that group 1 and 2 genes were enriched in the categories glycerolipid biosynthetic process, phospholipid biosynthetic process, glutamine biosynthetic process, auxin signal pathway and chromatin modification, and groups 3 and 4 were enriched in the categories sucrose metabolic process, cellulose biosynthetic process, cytoskeleton organization, secondary cell wall biogenesis and glucuronoxylan biosynthetic process (Figure 1C). This unbalanced distribution of biological process reflects the different physiological events that occur during fiber elongation.

### Cluster analysis of differentially expressed genes between and/or among CSILs

To identify differentially expressed genes (DEGs) in the CSILs, we examined 20 comparison groups between CSILs and TM-1 from 5 DPA to 20 DPA. The number of DEGs between the same developmental stage ranged from 4,500 to 8,000 (Additional file 2: Figure S1). However, the number of DEGs was lower in some libraries than in others, especially in CSIL-35431 and CSIL-35368 at 5 DPA and in CSIL-31044 at 15 DPA. Interestingly, we found that more genes were upregulated than downregulated throughout the elongation stage in CSIL-35431.

**Figure 2:**
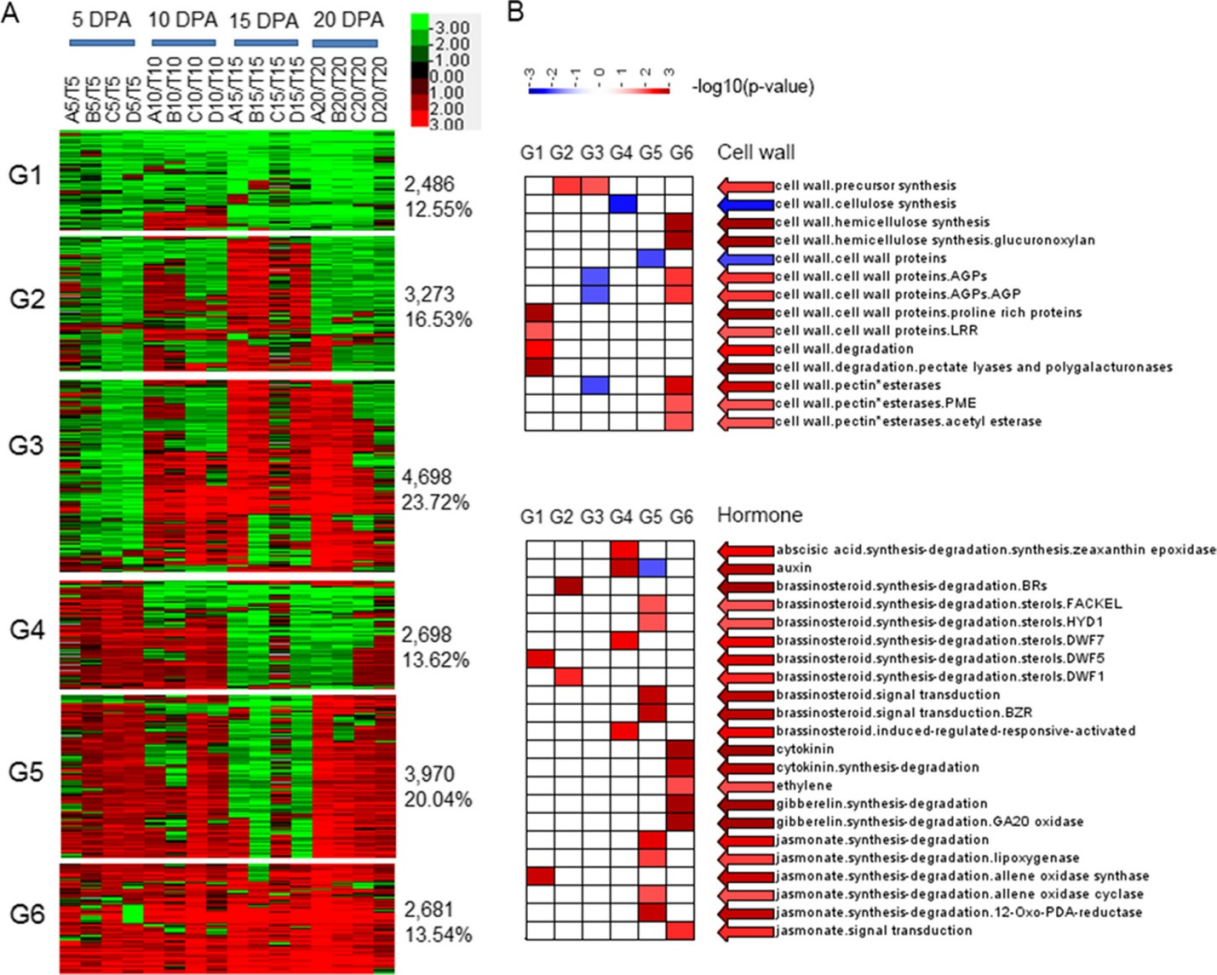
**Cluster and functional enrichment analysis of DEGs in four longer fiber CSILs. (A)** Cluster analysis of DEGs in four CSILs with superior fiber quality compared to TM-1. A, B, C, D and T indicate CSIL-35431, CSIL-31134, CSIL-31068, CSIL-31044 and TM-1, respectively. 5, 5 DPA; 10, 10 DPA; 15, 15 DPA; 20, 20 DPA. Red indicates upregulated genes and green indicates downregulated genes. **(B)** Gene enrichment analysis of DEGs involved in cell walls and hormones. Colors from blue to red indicate that functions were enriched more significantly with smaller p-values.

To examine the expression patterns of the DEGs, we performed cluster analysis of 19,806 DEGs expressed in four CSILs. These DEGs were grouped into six clusters according to their expression patterns, designated G1–G6 (Figure 2A). Compared to TM-1, 2,486 genes in the G1 category had low expression levels from 5 to 20 DPA, while 3,273 genes in G2 were highly expressed at 10 DPA and 20 DPA, 4,698 genes in G3 were highly expressed from 10 to 20 DPA, 2,698 genes in G4 were highly expressed at 5 DPA and 10 DPA, 3,970 genes were highly expressed at 5 DPA, 10 DPA and 20 DPA, and 2,681 genes in G6 were highly expressed throughout the elongation stage.

### Gene functional annotation by GO enrichment

To identify possible biological pathways that were altered in the CSILs, GO functional enrichment was performed using an FDR adjusted p-value of ≤0.05 as the cutoff. The GO annotations of DEGs related to cell wall formation and hormone for all six groups are shown in Figure 2B. We found that DEGs enriched in cell wall formation were mainly in G1 and G6. In G1, DEGs involved in cell wall degradation were downregulated in CSILs, such as the categories pectate lyase and polygalacturonase. In G6, DEGs involved in hemicellulose synthesis, cell wall protein and pectin were upregulated in the CSILs, such as the categories glucuronoxylan, AGPs and pectin esterase. Plant hormones play an important role in fiber development. DEGs related to almost all known hormones including auxin, BRs, ABA and jasmonic acid were mainly in G4 and G5. DEGs related to ethylene, cytokinin and GAs were mainly in G6.

We also analyzed the functional enrichment of common DEGs in four longer fiber CSILs from 5 to 20 DPA and selected 424, 908, 493 and 956 common DEGs at 5, 10, 15 and 20 DPA, respectively (Additional file 3: Figure S2A). Although there were fewer common DEGs at 5 DPA and 15 DPA, more processes were enriched at these two stages, including major/minor CHO metabolism, glycolysis process, cell wall, lipid metabolism and secondary metabolism (Additional file 3: Figure S2B). In addition, GO enrichment analysis of common upregulated DEGs in these four CSILs showed that there were many DEGs associated with molecular functions related to carbohydrate metabolism (Additional file 4: Table S2).

To identify the possible biological process that led to shorter fibers in CSIL-35368, we examined DEGs that were downregulated only in CSIL-35368 from 5 to 20 DPA (Additional file 5: Figure S3). The significantly enriched GO terms included microtubule-base movement and nucleosome at 5 DPA, nucleosome assembly and starch biosynthetic process at 10 DPA, cellular lipid catabolic process and xylem vessel member cell differentiation at 15 DPA and wax biosynthetic process at 20 DPA. We found five genes that were downregulated in CSIL-35368 but upregulated in CSIL-35431, CSIL-31134 and CSIL-31068 from 5 to 25 DPA, including β-glucosidase 41 (Gorai.013G165100.1), expansin-like B1 (Gorai.004G192500.1) and transmembrane protein 97 (Gorai.008G173000.1) at 5 DPA, scarecrow-like 5 (Gorai.002G096300.1) at 10 DPA and phosphoinositide 4-kinase (Gorai.003G078600.1) and kinesin 13A (Gorai.011G190700.2) at 20 DPA. Additionally, we found DEGs upregulated only in CSIL-35368 were involved in cytokinin-signaling pathway (GO:0009736) at 10 DPA and negative regulation of cellular biosynthetic process (GO:0031327) at 20 DPA. These results indicate that the carbohydrate, cytoskeleton, wax, hormone and lipid processes play key roles in fiber elongation.

### Pathway analysis of DEGs in the CSILs

**Figure 3:**
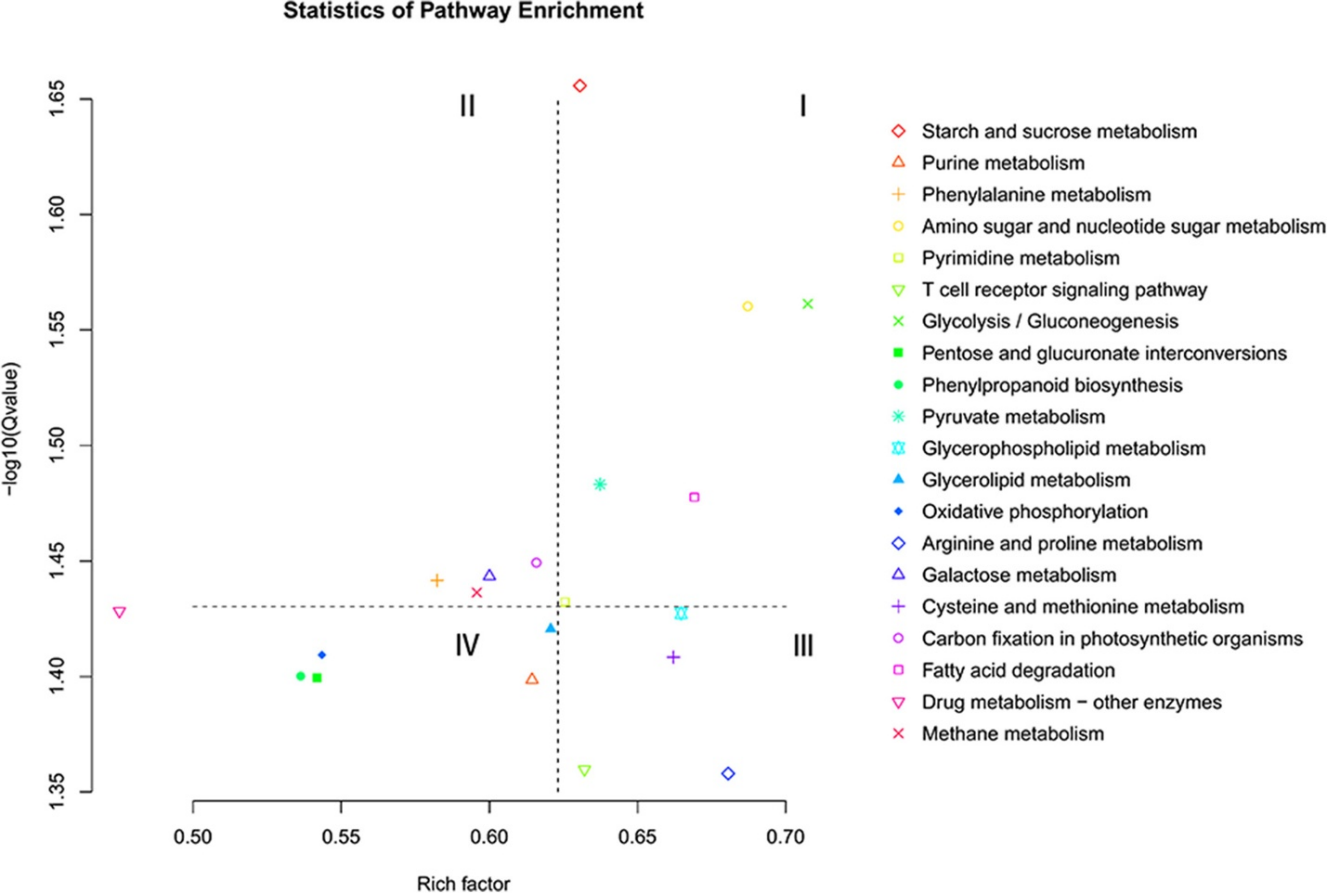
**Pathway analysis of DEGs in four longer fiber CSILs.** All DEGs in G1–G6 are used in pathway analysis according to Figure 2. Twenty pathways were selected by Q-value (corrected P-value). Rich factor indicates that the sample number/reference number participated in the same pathway. The order of significance was I > II > III > IV.

Based upon GO analysis, we determined that lots of biological processes were affected in the CSILs, but it was still not quite clear how the mechanisms underlying fiber elongation were affected in the CSILs. Therefore, we performed pathway analysis of 19,806 DEGs in the four CSILs, according to Figure 2. Notably, the most highly enriched pathways included starch and sucrose metabolism (355 genes), amino sugar metabolism (145 genes), glycolysis/gluconeogenesis (133 genes), pyruvate metabolism (116 genes) and fatty acid degradation (89 genes) (Figure 3). In addition, the pathways phenylalanine metabolism, galactose metabolism and oxidative phosphorylation were enriched; additional pathways are listed in Additional file 6: Table S3.

Pathway analysis of common DEGs (2,781 genes) showed similar results, according to genes in Additional file 3: Figure S2. The most highly enriched pathways were fatty acid biosynthesis (1.27E-02), glycolysis/gluconeogenesis (1.40E-04), methane metabolism (1.23E-05), starch and sucrose metabolism (1.10E-03) and others (Additional file 7: Figure S4 and Additional file 8: Table S4).

### The expression of hormone-, cell wall-, lipid metabolism- and transcription factor-related genes was substantially altered in the CSILs

**Figure 4:**
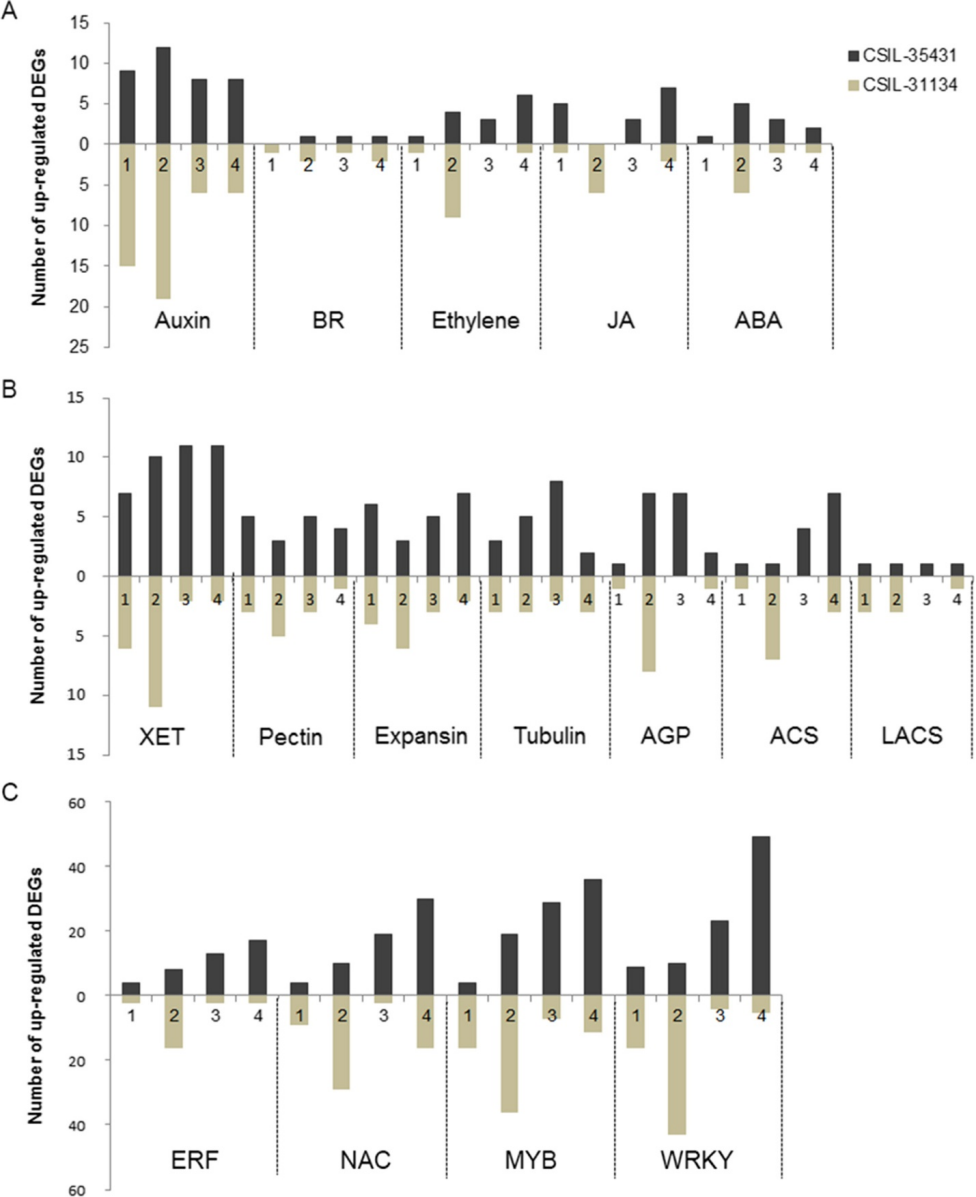
**Distribution of upregulated DEGs during the elongation stage. (A)** Number of upregulated DEGs response to hormone including auxin, BR, ethylene, JA and ABA. **(B)** Number of upregulated DEGs involved in primary cell wall biosynthesis. XET, xyloglucan endotransglucosylase; AGP, arabinogalactan; ACS, ACC synthase; LACS, long-chain acyl-CoA synthetase; pectin included pectin methylesterase and pectin acetylesterase. **(C)** Number of upregulated DEGs related to transcription factors. Numbers 1–4 in axis represent 5 DPA, 10 DPA, 15 DPA and 20 DPA, respectively. Black bar indicates CSIL-35431 and gray bar indicates CSIL-31134.

Phytohormones play an important regulatory role in various plant growth and developmental processes. In the present study, DEGs involved in hormone biosynthesis and signal transduction pathways were identified in the CSILs. Numerous genes involved in hormone signal transduction and biosynthesis of auxin, BR, ethylene, JA and ABA were upregulated in the CSILs (Figure 4A and Additional file 9: Table S5). In both CSIL-35431 and CSIL-31134, the expression of genes involved in auxin was more dramatically altered than that of other hormones. There were more such upregulated genes in CSIL-35431 than in CSIL-31134 throughout the elongation stage, except at 10 DPA. Notably, more genes related to all five hormones were upregulated at 10 DPA.

Several genes involved in cell wall and fatty acid biosynthesis were differentially expressed at various stage of fiber elongation in the CSILs compared to TM-1 (Figure 4B and Additional file 9: Table S5). More genes, such as genes involved in xyloglucan endotransglycosylases (XETs), arabinogalactan (AGP) and pectin (including pectin methyl esterase [PME] and pectin acetyl esterase 1-4), expansin and tubulin, were upregulated in CSIL-35431 than in CSIL-31134. However, more ACC synthase (ACS) and long-chain acyl-CoA synthetase (LACS) genes were upregulated in CSIL-31134 than in CSIL-35431. Similar to hormone-related genes, at the early stages, the expression of the above genes was distinctly altered at 5 DPA and 15 DPA in CSIL-31134.

Moreover, genes involved in transcription factors including ERF, NAC, MYB and WRKY were highly upregulated in the CSILs (Figure 4C and Additional file 9: Table S5). These genes in CSIL-35431 were differentially expressed at the later elongation stage, while an opposite result was obtained for CSIL-31134.

It was interesting that most of these genes were differentially expressed at 10 DPA in CSIL-31134. However, in CSIL-35431, these genes were upregulated at early stage, and TFs were upregulated at 15 DPA and 20 DPA in CSIL-35431. Our result indicated that metabolic pathways were differentially altered in CSIL-35431 vs. CSIL-31134.

### Metabolism associated with fiber elongation

Sugars represent a basic source of energy, and they provide carbon skeletons for all biomolecules and are required for the regulation of cell homeostasis and the synthesis of cell wall precursors. Our result show that genes involved in minor CHO (carbohydrate), cell wall, lipid, starch and sucrose metabolism exhibited altered expression at 10 DPA in all three CSILs (Additional file 10: Figue S5). In particular, CSIL-31134 had more DEGs involved in metabolism, and most DEGs encoding cell wall proteins and cellulose synthase showed upregulated expression in CSIL-35431. In addition, numerous genes associated with minor CHO metabolism were downregulated in CSIL-35368, especially callose-related genes.

**Figure 5:**
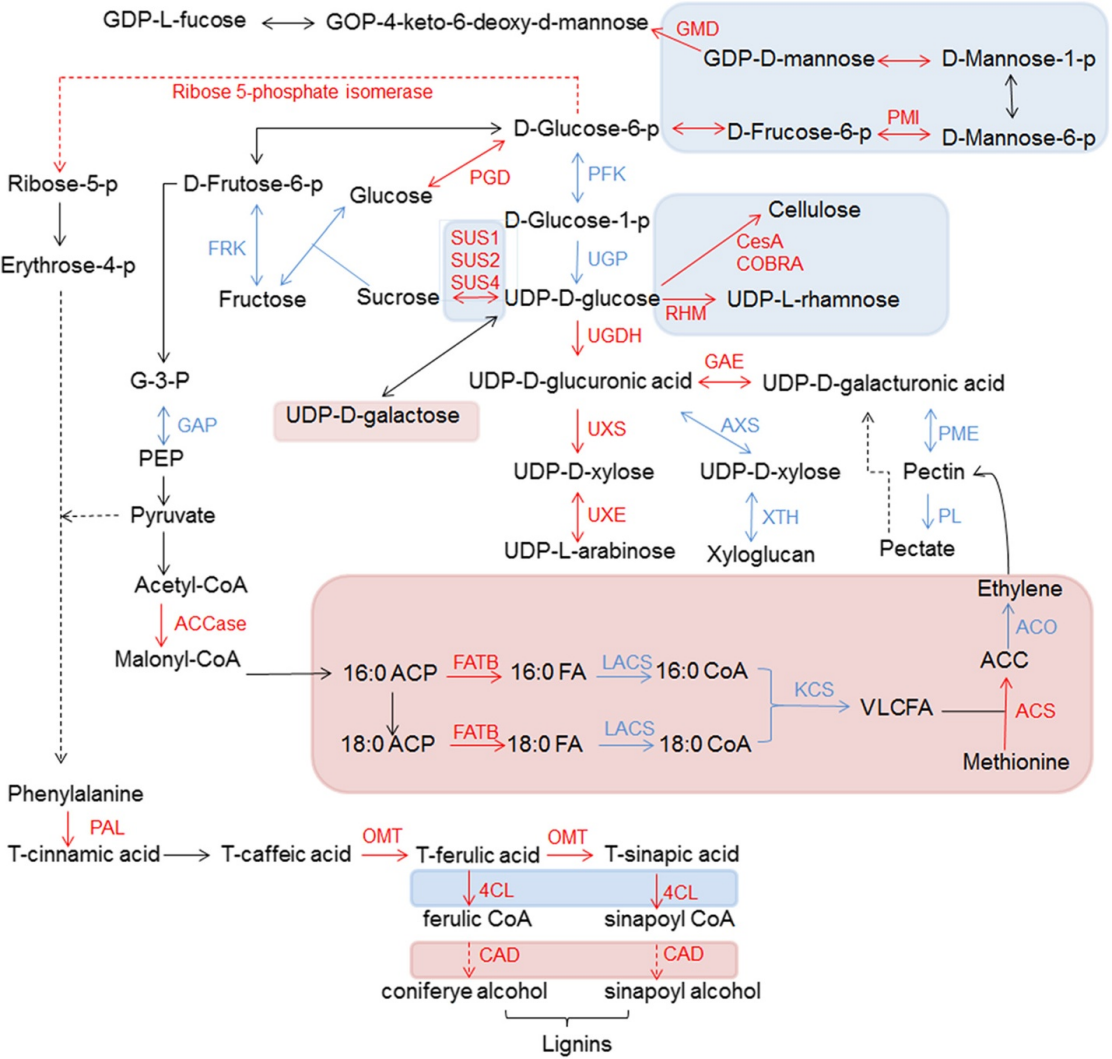
**Metabolic pathways for carbohydrates, fatty acids and phenylalanine.** All genes are selected based on CSIL-35431 at 10 DPA. Red text and line indicate upregulated DEGs, while blue text and line indicate downregulated DEGs. Blue shaded box indicates that the pathway was only upregulated or altered more significantly in CSIL-35431, and pink shaded box indicates that the pathway is altered more significantly in CSIL-31134. ACCase, acetyl-CoA carboxylase; ACP, acyl carrier protein; ACC, 1-aminocyclopropane-1-carboxylic acid; ACO, ACC oxidase; ACS, ACC synthase; AXS, UDP-D-xylose synthase; CAD, cinnamyl alcohol dehydrogenase; CESA, cellulose synthase; 4CL, 4-coumarate CoA ligase; FATB, fatty acyl-ACP thioesterases B; FRK, fructokinase; GAE, UDP-D-glucuronate 4- epimerase; GAP, glyceraldehyde-3-phosphate; GMD, GDP-mannose 4,6-dehydratase; KCS, 3-ketoacyl-CoA synthase; LACS, long-chain acyl-CoA synthetase; OMT, o-methyl transferase; PAL, phenylalanine ammonia lyase; PFK, phosphofructokinase; 6-PGD, 6-phosphogluconate dehydrogenase; PL, pectin lyase; PME, pectin methylesterase; PMI, mannose-6-phosphate isomerase; RHM, rhamnose synthase; SUS, sucrose synthase; UGDH, UDP-glucose 6-dehydrogenase; UGP, UDP-glucose pyrophosphorylase; UXE, UDP-arabinose 4-epimerase; UXS, UDP-glucuronate decarboxylase; VLCFA, very long-chain fatty acid; XTH, xyloglucan endotransglucosylase.

Regulatory networks showed genes involved in cell wall precursors, glycolysis, pentose phosphate, fatty acid and phenylpropanoid metabolism were mostly upregulated in the CSILs (Figure 5). Pyruvate and acetyl-CoA are products of glycolysis and are related to VLCFA biosynthesis and phenylpropanoid metabolism, and very long-chain fatty acid (VLCFA) is linked to ethylene biosynthesis. Genes involved in encoding nucleotide sugars such as fucose, rhamnose, arabinose and sucrose, cell wall precursors, pectin and cellulose biosynthesis were more significantly upregulated in CSIL-35431, while galactose, VLCFA and ethylene biosynthesis genes were more significantly upregulated in 31134. In addition, genes encoding 4CL and CAD (in the phenylpropanoid metabolic pathway) were upregulated in different stages in CSIL-35431 and CSIL-31134, respectively. In other CSILs, different expression pattern of DEGs involved in fucose, xylose and lignin biosynthesis were showed in Additional file 11: Table S6. These results suggest that at 10 DPA, differences in metabolism may affect fiber elongation in the CSILs.

**Figure 6:**
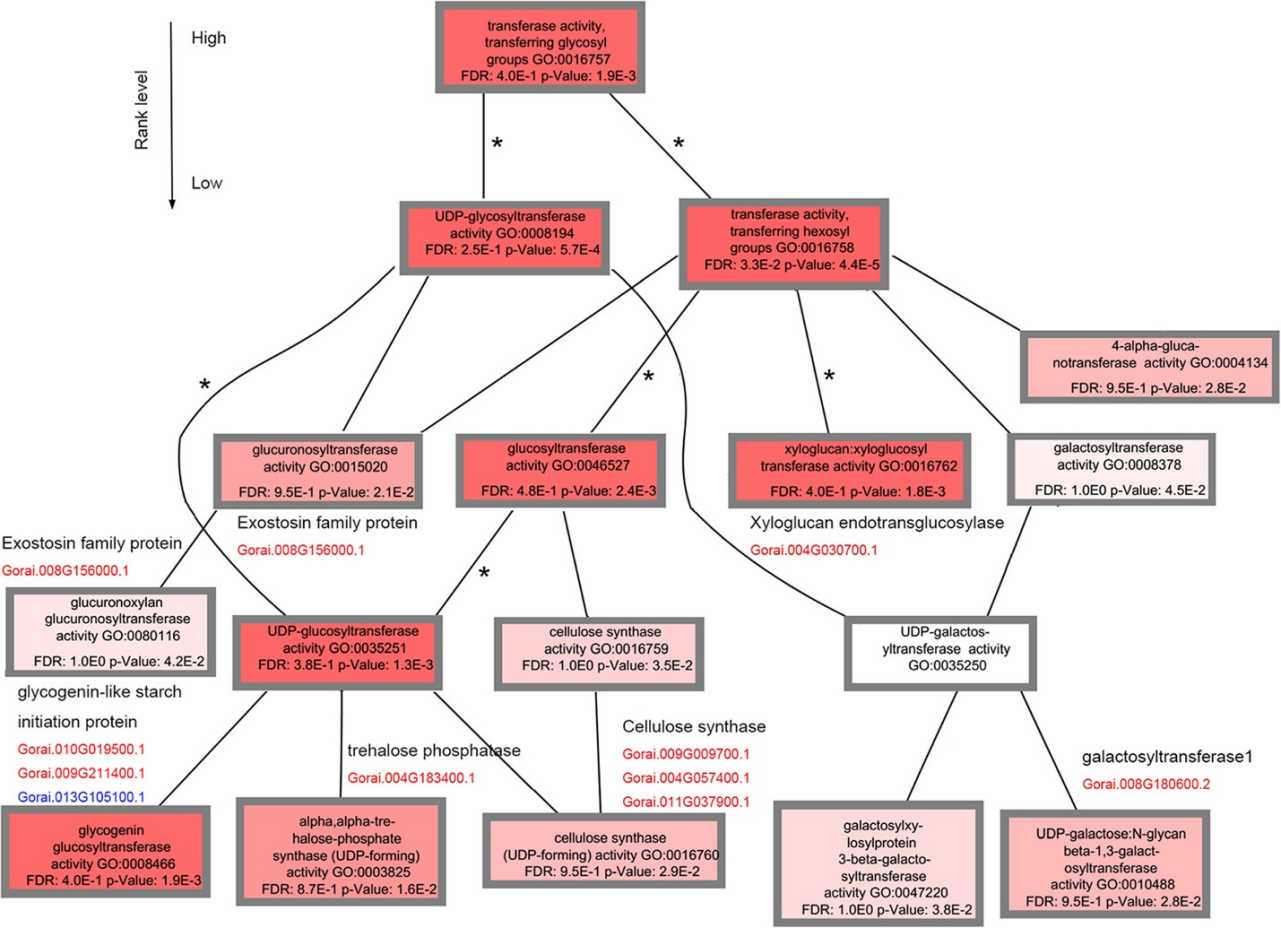
**GO term graph of common DEGs in four CSILs at 15 DPA.** These common genes are according to Figure S2. Each square indicates one molecular function with GO term number. The GO terms level decreased from top to bottom. As the color deepens (from white to red), the molecular functions were enriched more significantly, with smaller P-values. Each gene family in a GO term is listed in black. DEGs indicated in red were upregulated in four CSILs, and DEGs indicated in blue were upregulated in three CSILs. The line with an asterisk indicates that the same molecular function is enriched, using common DEGs at 10 DPA.

To further examine genes related to carbohydrate metabolism, we constructed a directed acyclic graph using common DEGs involved in glycosyltransferase activity (GO: 0016757) at 10 DPA and 15 DPA in the four longer fiber CSILs (Figure 6). Almost all of the GO terms were enriched at 15 DPA, and six GO terms (asterisk marker in the graph) were enriched at 10 DPA. Genes encoding proteins such as xyloglucan endotransglycosylases (XET), glycogenin-like starch initiation protein (GUX), galatosyltransferase and others may play important roles in fiber elongation.

### Mining of DEGs in introgressed *G. barbadense*chromosome segments

According to the locations of introgressed *G. barbadense* chromosome segments detected in a previous study [37], we searched the genome sequence of *G. raimondii* [45], selected related genes located in these segments and identified candidate genes comparing the DEGs from the transcriptome data using the same gene name. Fortunately, two genes in CSIL-35431, 12 genes in CSIL-31134 and 56 genes in CSIL-31044 exhibited upregulated expression in the transcriptome data during the fiber elongation stage. In addition, most of these confirmed genes were upregulated mainly at 10 DPA (Additional file 12: Table S7). ARM repeat and protein kinase superfamily genes showed upregulated expression in CSIL-35431, and upregulated genes encoding amino acid kinase family and zinc finger proteins were detected in CSIL-31134. Moreover, functional enrichment analysis of the 56 upregulated genes in CSIL-31044 indicated that genes involved in transferase activity, including glucuronosyltransferase activity (GO:0080116), inositol-1, 4, 5-trisphosphate 3-kinase activity (GO:0008440) and desulfoglucosinolate sulfotransferase activity (GO:0047364), were enriched in CSIL-31044 (Additional file 13: Figure S6); the results of enrichment analysis are similar to the results of transcriptome analysis of DEGs in the CSILs.

To validate the transcriptome results, we selected 13 genes in two plant materials for quantitative real-time PCR analysis. The results of pair-by-pair comparisons between the transcriptome data and qRT-PCR analysis and qRT-PCR primers are shown in Additional file 14: Table S8. The expression levels were highly correlated (r^2^ = 0.75 - 0.99); therefore, quantitative real-time PCR confirmed the accuracy and reliability of the expression levels determined by DGE analysis.

## Discussion

Cotton fiber cell elongation is a complex and highly regulated process involving metabolic pathways, signal transduction and transcriptional regulation. To date, the roles of carbohydrate metabolism, phytohormones, lipid metabolism and transcription factors in promoting cotton fiber elongation have been underexplored. CSILs with longer fibers provide an excellent system for studying cotton fiber elongation. First, our results support the notion that these metabolic and signaling pathways are significantly upregulated and cooperate during cotton fiber elongation. In addition, we obtained detailed information about DEGs involved in cell wall precursor biosynthesis (fucose, rhamnose, galactose, arabinose and callose biosynthesis), cell wall-related protein (AGP, LRR), very-long-chain fatty acid biosynthesis (ACCase, FATB, KCS), phenylpropanoid pathway (OMT, 4CL, CAD) and so on. We also identified new candidate genes in introgressed segments by combing the transcriptome data. Our integrated analysis provided additional details and new insights into the mechanisms of fiber elongation.

### Carbohydrate metabolism, cell wall-loosening and cytoskeleton genes associate with fiber cell elongation

Function enrichment, KEGG analysis and metabolic pathway analysis showed that the categories carbohydrate metabolism and cell wall biosynthesis were highly enriched in the CSILs, and the categories cell wall processor synthesis, cellulose synthesis, hemicellulose synthesis, cell wall proteins (AGP, LRR) and pectin were enriched as well (Figure 2, 3 and Additional file 10: Figure S5). We also identified a large number of genes involved in primary cell wall biosynthesis and elongation, such as xyloglucan endotransglycosylases, pectin modification enzymes, expansins, tubulins and arabinogalactans that were upregulated in the CSILs during fiber elongation (Figure 4 and Additional file 9: Table S5). Previous studies have shown that xyloglucan modifying enzymes such as xyloglucan endotransglycosylases (XETs) play a role in fiber cell development [22, 23, 46]. In the current study, XET22, XET28 and other XETs were highly upregulated in the CSILs during fiber elongation, which was similar to previous reports [46]. In particular, more XETs were upregulated in CSIL-35431 from 5 to 20 DPA.

Cellulose synthase genes were significantly upregulated in CSIL-35431, such as genes encoding CESA3, CESA6 and CESA7. In *G. raimondii*, at least 15 cellulose synthase (CESA) genes are required for cellulose synthesis [45]. Recently, six full-length CESA genes (designated *GhCESA5–GHCESA10*) were identified; CSEA1, CESA3, CESA6, CESA9 and CESA10 are involved in primary cell wall biosynthesis [47]. In addition, *COBRA* is critical for the biosynthesis of cell wall components [48, 49].

Pectin is a polysaccharide component of the primary cell wall, and pectin modification enzymes play an important role in fiber elongation [20, 22, 50]. We examined the expression of genes encoding two types of pectin modification enzymes, such as pectin methyl esterase (PME) and pectin acetyl esterase, which were upregulated in CSILs from 5 to 20 DPA. In CSIL-31134, more pectin modification enzymes were upregulated during the early stage of cotton fiber development than.

Arabinogalactan proteins (AGPs), which are involved in many aspects of plant development, are abundant in developing fibers and are involved in fiber elongation. *GhFLA1* improves fiber initiation and elongation by affecting the integrity of the primary cell wall matrix [51]. We found that AGPs genes were upregulated mainly at 10 DPA in CSIL-35431 and CSIL-31134, with high levels of increased expression (Figure 4B). Similar results were obtained from metabolic pathway analysis (Additional file 10: Figure S5).

Expansins and tubulins are important for cell wall-loosening and fiber elongation [25, 26, 46, 52, 53]. Expansins comprise four subfamilies including α-Expansin (EXPA), β-Expansin (EXPB), Expansin-like A (EXLA) and Expansin-like B (EXLB) [54]. EXPAs produce polysaccharide complexes such as xyloglucan and pectin, which link cellulosic microfibrils together [55]. In the current experiment, Expansin A1 (α-expansin 1) genes were highly expressed in the CSILs, while one expansin-like B1 gene was only upregulated in CSIL-35431. In addition, the β-tubulin 6 gene was only upregulated in CSIL-35431 during the early stage of cotton fiber elongation.

Mining of DEGs in introgressed *G. barbadense* segments also showed that genes involved in glucuronosyltransferase, inositol-1, 4, 5-trisphosphate 3-kinase and desulfoglucosinolate sulfotransferase (SOT) activity were upregulated in the CSILs, indicating the important roles of these enzymes in carbohydrate metabolism. Several SOT proteins have been characterized in *Flaveria* sp. and *Brassica napus* L. that show substrate specificity for several flavonols, steroids and brassinosteroids, and these genes were involved in various physiological processes, such as growth, development and adaptation to stress [56–58]. Also, steroid sulfotransferases are targeted by small RNAs in fiber initials [59].

### Fatty acids and secondary metabolic pathways associated with fiber cell elongation

Fatty acids are another important factor involved in fiber elongation [60, 61]. Several ACC synthase and long-chain acyl-CoA synthetase genes were upregulated in the CSILs, and more DEGs associated with metabolic pathways were detected (Figure 5 and Additional file 10: Figure S5). KEGG and metabolic pathway analyses also identified numerous DEGs that participate in fatty acid metabolism. For example, ACCase, FATB, LACS and KCS genes, which are important for VLCFA biosynthesis, were upregulated in CSIL-31134 [62].

Secondary metabolism-related genes were among the most statistically significant differentially expressed categories between CSILs and TM-1 during fiber elongation. The phenylpropanoid pathway participates in the biosynthesis of many plant cell wall phenolics, which are responsible for the biosynthesis of a variety of products including lignins, lignans, hydroxycinnamic acid conjugates, flavonoids and other related constituents, and cinnamyl alcohol dehydrogenase (CAD) is considered to be a key enzyme in the biosynthesis of these products [63]. Our results show that 4CL and CAD genes were upregulated in CSIL-35431 and CSIL-31134, respectively. The expression of phenylpropanoid genes was highly correlated with specific fiber properties in an inter-specific cotton recombinant inbred line (RIL) population, supporting their role in determining fiber quality [64].

### Phytohormones and TFs associated with fiber cell elongation

In this study, a large group of genes response to hormones including auxin, BR, ethylene, JA and ABA were upregulated in the CSILs, which were shown to play roles in fiber cell elongation (Figure 4 and Additional file 11: Table S6) [13, 18, 20]. We found that more auxin response genes were upregulated during the early stage (5 and 10 DPA) than during the later stage (15 and 20 DPA) of fiber development, including auxin response factor genes and SAUR-like auxin responsive genes. In addition, the enrichment results show that genes involved in auxin signaling pathways were more highly expressed at the early stage, as previously reported [7]. Also, we found that nine ethylene responsive element binding factor genes had high expression levels in the CSILs in the early stage of fiber elongation; ethylene is an important factor in fiber elongation [11]. We also detected three upregulated BR genes in the CSILs; BR biosynthesis can induce the expression of *GhTUB1*, *GhTUB3* and *GhTUB9* in cultured cotton ovules [65].

TF genes belonging to the ERF, NAC, MYB and WRKY families were upregulated, and the number of upregulated DEGs differed in CSIL-35431 and CSIL-31134. In CSIL-31134, most upregulated TF genes were detected at 10 DPA, including 16 ERF genes, 29 NAC genes, 36 MYB genes and 43 WRKY genes (Additional file 9: Table S5). MYB family genes, such as *GhMYB4*, *GhMYB6*, *GhMYB109* and *GhMYB25*, are associated with fiber cell elongation or trichome development [15, 28, 29, 66–68].

### Differentially altered metabolic pathway genes in the CSILs

Interestingly, by performing PCC analysis of all 24 libraries, we determined that different PCC values existed between the CSILs and TM-1, particularly at 15 DPA and 20 DPA (Figure 1). Normally, a low PCC value was observed between different stages only in TM-1 as a result of altered expression levels of most genes during fiber elongation. We propose that the expression of DEGs in different metabolic pathways is altered differentially in these lines, particularly during the later stage of fiber elongation.

Several metabolic pathways were also examined to help uncover the mechanism of altered fiber elongation, including cell wall, lipids, minor CHO, starch and sucrose pathways (Additional file 10: Figure S5). The number of DEGs in CSIL-35431, CSIL-31134 and CSIL-35368 differed at 10 DPA, with more DEGs identified in CSIL-31134. In CSIL-35431, most DEGs encoding cell wall protein (AGP, LRR), callose and cellulose (CESA, COBRA) and genes involved in cell wall precursor synthesis were upregulated. Fatty acid and ethylene-related genes were upregulated in CSIL-31134. In CSIL-35368, numerous downregulated genes were involved in lipid metabolism and starch and sucrose metabolism, especially in callose (Additional file 10: Figure S5). Callose serves as a matrix for the deposition of other cell wall materials and as a cell wall-strengthening material in cotton fibers and growing pollen tubes [10, 69]. Our functional enrichment results also show that DEGs downregulated only in CSIL-35368 were enriched in microtubule-base movement, lipid catabolic process, wax biosynthetic in other stages; these categories were reported to be related to fiber elongation.

## Conclusions

**Figure 7:**
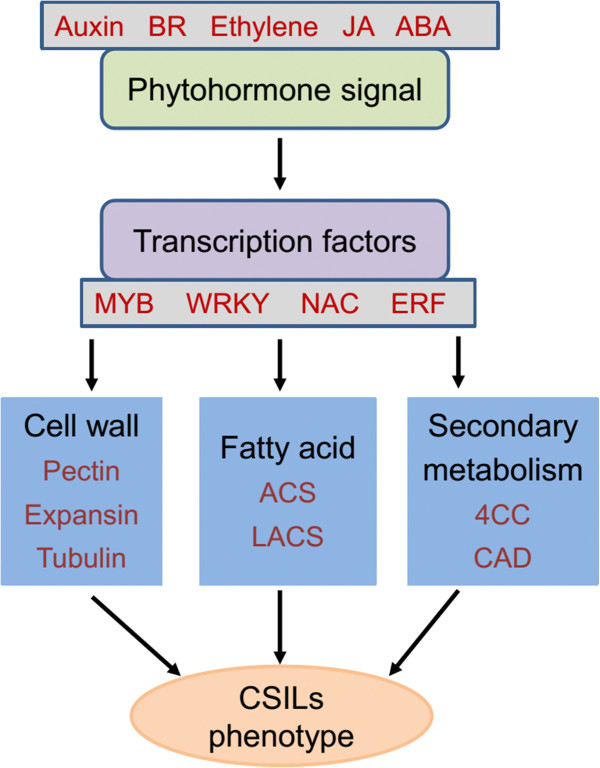
**Integrative network for the mechanism of fiber elongation in CSILs.** This network includes hormone responses, transcription factors, cell wall biosynthesis, fatty acid metabolism and secondary metabolism. Red text notes related genes within these pathways.

Based on the results from transcriptome comparison of the CSILs to recurrent parent, we established an integrative network to elucidate the mechanism of fiber elongation in CSILs consistent with fiber quality (Figure 7). Functional enrichment and metabolic pathway analysis provided important information about the molecular mechanisms controlling fiber length, which are mainly tied to carbohydrate metabolism, cell wall biosynthesis, fatty acid metabolism, secondary metabolism, hormone responses and TFs. We proposed that expression of genes involved in cell wall biosynthesis, fatty acid metabolism and secondary metabolism were changed in CSILs as a result of altered regulation of phytohormone signal pathways and transcription factors, which were commonly identified as being involved in regulating elongation stages. Additionally, metabolic pathways were differentially altered in CSILs harboring different *G. barbadense* chromosome segments, which indicate that these integrated metabolic pathways result in the production of extra-long staple fibers in *G. barbadense*, and modifying these different pathways can potentially improve fiber qualities in *G. hirsutum*.

### Accession numbers

All the raw data supporting the results of this article have been deposited at the DDBJ/EMBL/GenBank short read archives (SRA) under the accession number SRP047139.

## Electronic supplementary material

Additional file 1: Table S1: Categorization and abundance of tags. (XLS 36 KB)

Additional file 2: Figure S1: Statistics of DEGs between CSILs and TM-1 from 5 DPA to 20 DPA. Orange bar, upregulated genes compared to TM-1; blue bar, downregulated genes compared to TM-1, green square, total DEGs. CSILs included CSIL-35431, CSIL-31134, CSIL-31068, CSIL-31044 and CSIL-35368, compared with TM-1. 5, 5 DPA; 10, 10 DPA; 15, 15 DPA; 20, 20 DPA. (TIFF 248 KB)

Additional file 3: Figure S2: Functional enrichment analysis of common DEGs between four CSILs with superior fiber quality. (A) Common DEGs between four CSILs from 5 DPA to 20 DPA. A, B, C, D indicated CSIL-35431, CSIL-31134, CSIL-31068, CSIL-31044, respectively. (B) Gene enrichment analysis of common DEGs. Color from blue to red means function enriched with smaller p-value. (TIFF 491 KB)

Additional file 4: Table S2: GO enrichment analysis of common upregulated DEGs in four CSILs. (XLS 34 KB)

Additional file 5: Figure S3: GO enrichment analysis of genes downregulated only in CSIL-35368. (A) Common DEGs of four CSILs. A, B, C, D indicated CSIL-35431, CSIL-31134, CSIL-31068, CSIL-35368, respectively. 5, 5DPA; 10, 10 DPA; 15, 15 DPA; 20, 20 DPA. (B) GO enrichment of DEGs only down-regulated in CSIL-35368 from 5 to 20 DPA. (TIFF 464 KB)

Additional file 6: Table S3: Pathway analysis of all DEGs in four CSILs with superior fiber quality. (XLS 215 KB)

Additional file 7: Figure S4: Pathway analysis of common DEGs between four CSILs from 5 to 20 DPA. All the DEGs used to analysis pathway were according to Figure 2. Twenty pathways were selected by Q-value (corrected P-value). Rich factor means the sample number/reference participated in the same pathway. Significance wasI > II > III > IV. (TIFF 130 KB)

Additional file 8: Table S4: Pathway analysis of common DEGs between four CSILs. (XLS 72 KB)

Additional file 9: Table S5: Expression levels of DEGs involved in hormones, cell wall biosynthesis and TFs. (XLS 144 KB)

Additional file 10: Figure S5: Overview of metabolism-related DEGs in different CSILs at 10 DPA. (A) Overview of metabolism-related DEGs in CSIL-35431. (B) Overview of metabolism-related DEGs in CSIL-31134. (C) Overview of metabolism-related DEGs in CSIL-35368.1, cell wall proteins, HRGP; 2, cell wall proteins, AGPs; 3, cell wall proteins, LRR; 4, minor CHO metabolism, callose; 5, cell wall precursor synthesis; 6, cell wall pectin esterases and PME; 7, cellulose synthesis; 9, FA synthesis and FA elongation. 10, starch synthesis; 11, starch degradation; 12, sucrose degradation. Blue square, downregulated gene; Red square, upregulated gene. (TIFF 970 KB)

Additional file 11: Table S6: List of DEGs that participate in different metabolic pathways. (XLS 48 KB)

Additional file 12: Table S7: Upregulated DEGs located in introgressed *G. barbadense* chromosome segments. (XLS 42 KB)

Additional file 13: Figure S6: GO term graph of upregulated DEGs located in introgressed *G. barbadense* chromosome segments. Every square indicated one molecular function with GO term number. From up to down, the rank level of GO term became lower. With the color deepened, from white to red, the molecular functions were enriched more significantly with smaller P-value. DEGs involved the GO term were listed. (TIFF 305 KB)

Additional file 14: Table S8: Correlation between quantitative real-time PCR and DGE analysis of 13 DEGs. (XLS 30 KB)

## Abbreviations

CSIL: Chromosome segment introgression line
DEG: Differentially expressed gene
DGE: Digital gene expression
DPA: Days post-anthesis
GO: Gene ontology
KEGG: Kyoto encyclopedia of genes and genomics
PCC: Pearson correlation coefficient
TPM: Transcripts per million.

## References

[1] Arpat AB, Waugh M, Sullivan JP, Gonzales M, Frisch D, Main D, Wood T, Leslie A, Wing RA, Wilkins TA (2004). Functional genomics of cell elongation in developing cotton fibers. Plant Mol Biol.

[2] Wilkins TA, Arpat AB (2005). The cotton fiber transcriptome. Physiol Plantarum.

[3] Basara AS, Malik CP (1984). Development of cotton fiber. Inter Rev Cyto.

[4] Haigler TA, Jernstedt JA, Basra AM (1999). Molecular genetics of developing cotton fibers. Cotton Fibers.

[5] Kim HJ, Triplett BA (2001). Cotton fiber growth in planta and in vitro. Models for plant cell elongation and cell wall biogenesis. Plant Physiol.

[6] Lee JJ, Hassan OS, Gao W, Wei NE, Kohel RJ, Chen XY, Payton P, Sze SH, Stelly DM, Chen ZJ (2006). Developmental and gene expression analyses of a cotton naked seed mutant. Planta.

[7] Lee JJ, Woodward AW, Chen ZJ (2007). Gene expression changes and early events in cotton fibre development. Ann Bot-London.

[8] Ji S, Lu Y, Li J, Wei G, Liang X, Zhu Y (2002). A beta-tubulin-like cDNA expressed specifically in elongating cotton fibers induces longitudinal growth of fission yeast. Biochem Bioph Res Co.

[9] John ME, Keller G (1996). Metabolic pathway engineering in cotton: biosynthesis of polyhydroxybutyrate in fiber cells. Proc Natl Acad Sci U S A.

[10] Samuel Yang S, Cheung F, Lee JJ, Ha M, Wei NE, Sze SH, Stelly DM, Thaxton P, Triplett B, Town CD, Jeffrey Chen Z (2006). Accumulation of genome-specific transcripts, transcription factors and phytohormonal regulators during early stages of fiber cell development in allotetraploid cotton. Plant J.

[11] Shi YH, Zhu SW, Mao XZ, Feng JX, Qin YM, Zhang L, Cheng J, Wei LP, Wang ZY, Zhu YX (2006). Transcriptome profiling, molecular biological, and physiological studies reveal a major role for ethylene in cotton fiber cell elongation. Plant Cell.

[12] Tu LL, Zhang XL, Liang SG, Liu DQ, Zhu LF, Zeng FC, Nie YC, Guo XP, Deng FL, Tan JF, Xu L (2007). Genes expression analyses of sea-island cotton (*Gossypium barbadense* L.) during fiber development. Plant Cell Rep.

[13] Liu K, Sun J, Yao LY, Yuan YL (2012). Transcriptome analysis reveals critical genes and key pathways for early cotton fiber elongation in Ligon lintless-1 mutant. Genomics.

[14] Naoumkina M, Hinchliffe DJ, Turley RB, Bland JM, Fang DD (2013). Integrated metabolomics and genomics analysis provides new insights into the fiber elongation process in Ligon lintless-2 mutant cotton (*Gossypium hirsutum* L.). BMC Genomics.

[15] Wu Y, Machado AC, White RG, Llewellyn DJ, Dennis ES (2006). Expression profiling identifies genes expressed early during lint fibre initiation in cotton. Plant Cell Physiol.

[16] Guan XY, Chen ZJ (2011). Auxin boost for cotton. Nat Biotechnol.

[17] Zhang M, Zheng X, Song S, Zeng Q, Hou L, Li D, Zhao J, Wei Y, Li X, Luo M, Xiao Y, Luo X, Zhang J, Xiang C, Pei Y (2011). Spatiotemporal manipulation of auxin biosynthesis in cotton ovule epidermal cells enhances fiber yield and quality. Nat Biotechnol.

[18] Luo M, Xiao Y, Li X, Lu X, Deng W, Li D, Hou L, Hu M, Li Y, Pei Y (2007). *GhDET2*, a steroid 5 alpha-reductase, plays an important role in cotton fiber cell initiation and elongation. Plant J.

[19] Sun Y, Veerabomma S, Abdel-Mageed HA, Fokar M, Asami T, Yoshida S, Allen RD (2005). Brassinosteroid regulates fiber development on cultured cotton ovules. Plant Cell Physiol.

[20] Gou JY, Wang LJ, Chen SP, Hu WL, Chen XY (2007). Gene expression and metabolite profiles of cotton fiber during cell elongation and secondary cell wall synthesis. Cell Res.

[21] Pang CY, Wang H, Pang Y, Xu C, Jiao Y, Qin YM, Western TL, Yu SX, Zhu YX (2010). Comparative proteomics indicates that biosynthesis of pectic precursors is important for cotton fiber and *Arabidopsis* root hair elongation. Mol Cell Proteomics.

[22] Padmalatha KV, Patil DP, Kumar K, Dhandapani G, Kanakachari M, Phanindra ML, Kumar S, Mohan TC, Jain N, Prakash AH, Vamadevaiah H, Katageri IS, Leelavathi S, Reddy MK, Kumar PA, Reddy VS (2012). Functional genomics of fuzzless-lintless mutant of *Gossypium hirsutum* L. cv. MCU5 reveal key genes and pathways involved in cotton fibre initiation and elongation. BMC Genomics.

[23] Lee J, Burns TH, Light G, Sun Y, Fokar M, Kasukabe Y, Fujisawa K, Maekawa Y, Allen RD (2010). Xyloglucan endotransglycosylase/hydrolase genes in cotton and their role in fiber elongation. Planta.

[24] Li XB, Cai L, Cheng NH, Liu JW (2002). Molecular characterization of the cotton *GhTUB1* gene that is preferentially expressed in fiber. Plant Physiol.

[25] Li XB, Fan XP, Wang XL, Cai L, Yang WC (2005). The cotton *ACTIN1* gene is functionally expressed in fibers and participates in fiber elongation. Plant Cell.

[26] Wang J, Wang HY, Zhao PM, Han LB, Jiao GL, Zheng YY, Huang SJ, Xia GX (2010). Overexpression of a profilin (*GhPFN2*) promotes the progression of developmental phases in cotton fibers. Plant Cell Physiol.

[27] Guan X, Lee JJ, Pang M, Shi X, Stelly DM, Chen ZJ (2011). Activation of *Arabidopsis* seed hair development by cotton fiber-related genes. PLoS One.

[28] Machado A, Wu Y, Yang Y, Llewellyn DJ, Dennis ES (2009). The MYB transcription factor *GhMYB25* regulates early fibre and trichome development. Plant J.

[29] Walford SA, Wu Y, Llewellyn DJ, Dennis ES (2011). *GhMYB25-like*: a key factor in early cotton fibre development. Plant J.

[30] Wang P, Ding YZ, Lu QX, Guo WZ, Zhang TZ (2008). Development of *Gossypium barbadense* chromosome segment substitution lines in the genetic standard line TM-1 of *Gossypium hirsutum*. Chinese Sci Bull.

[31] Eshed Y, Zamir D (1995). An introgression line population of lycopersicon pennellii In the cultivated tomato enables the identification and fine mapping of yield-associated QTL. Genetics.

[32] Liu SB, Zhou RG, Dong YC, Li P, Jia JZ (2006). Development, utilization of introgression lines using a synthetic wheat as donor. Theor Appl Genet.

[33] Takai T, Nonoue Y, Yamamoto SI, Yamanouchi U, Matsubara K, Liang ZW, Lin HX, Ono N, Uga Y, Yano M (2007). Development of chromosome segment substitution lines derived from backcross between *indica* donor rice cultivar ‘*Nona bokra*’ and *japonica* recipient cultivar ‘*Koshihikari*’. Breeding Sci.

[34] Zhu W, Lin J, Yang D, Zhao L, Zhang Y, Zhu Z, Chen T, Wang C (2009). Development of chromosome segment substitution lines derived from backcross between two sequenced rice cultivars, *Indica* recipient 93-11 and *Japonica* donor nipponbare. Plant Mol Biol Rep.

[35] Kohel R, Richmond T, Lewis C (1970). Texas marker-1. Description of a genetic standard for *Gossypium hirsutum* L. Crop Sci.

[36] Pan J, Zhang T, Kuai B (1994). Studies on the inheritance of resistance to *Verticillium dahliae* in cotton. J Nanj Agr Univ.

[37] Wang P, Zhu Y, Song X, Cao Z, Ding Y, Liu B, Zhu X, Wang S, Guo W, Zhang T (2012). Inheritance of long staple fiber quality traits of *Gossypium barbadense* in *G. hirsutum* background using CSILs. Theor Appl Genet.

[38] Jiang JX, Zhang TZ (2003). Extraction of total RNA in cotton tissues with CTAB-acidic phenolic method. Cotton Sci.

[39] Wang QQ, Liu F, Chen XS, Ma XJ, Zeng HQ, Yang ZM (2010). Transcriptome profiling of early developing cotton fiber by deep-sequencing reveals significantly differential expression of genes in a fuzzless/lintless mutant. Genomics.

[40] Benjamini Y, Yekutieli D (2001). The control of the false discovery rate in multiple testing under dependency. Ann Stat.

[41] Herrero J, Valencia A, Dopazo J (2001). A hierarchical unsupervised growing neural network for clustering gene expression patterns. Bioinformatics.

[42] Klie S, Nikoloski Z (2012). The choice between mapman and gene ontology for automated gene function prediction in plant science. Front Genet.

[43] Kanehisa M, Araki M, Goto S, Hattori M, Hirakawa M, Itoh M, Katayama T, Kawashima S, Okuda S, Tokimatsu T, Yamanishi Y (2008). KEGG for linking genomes to life and the environment. Nucleic Acids Res.

[44] Pfaffl MW (2001). A new mathematical model for relative quantification in real-time RT-PCR. Nucleic Aacids Res.

[45] Paterson AH, Wendel JF, Gundlach H, Guo H, Jenkins J, Jin DC, Llewellyn D, Showmaker KC, Shu SQ, Udall J, Yoo MJ, Byers R, Chen W, Doron-Faigenboim A, Duke MV, Gong L, Grimwood J, Grover C, Grupp K, Hu G, Lee TH, Li J, Lin L, Liu T, Marler BS, Page JT, Roberts AW, Romanel E, Sanders WS, Szadkowski E (2012). Repeated polyploidization of *Gossypium* genomes and the evolution of spinnable cotton fibres. Nature.

[46] Ji SJ, Lu YC, Feng JX, Wei G, Li J, Shi YH, Fu Q, Liu D, Luo JC, Zhu YX (2003). Isolation and analyses of genes preferentially expressed during early cotton fiber development by subtractive PCR and cDNA array. Nucleic Acids Res.

[47] Li A, Xia T, Xu W, Chen TT, Li XL, Fan J, Wang RY, Feng SQ, Wang YT, Wang BR, Peng L (2013). An integrative analysis of four CESA isoforms specific for fiber cellulose production between *Gossypium hirsutum* and *Gossypium barbadense*. Planta.

[48] Li YH, Qian O, Zhou YH, Yan MX, Sun L, Zhang M, Fu ZM, Wang YH, Han B, Pang XM, Chen M, Li J (2003). BRITTLE CULM1, which encodes a COBRA-like protein, affects the mechanical properties of rice plants. Plant Cell.

[49] Roudier F, Fernandez AG, Fujita M, Himmelspach R, Borner GHH, Schindelman G, Song S, Baskin TI, Dupree P, Wasteneys GO, Benfey PN (2005). *COBRA*, an Arabidopsis extracellular glycosyl-phosphatidyl inositol-anchored protein, specifically controls highly anisotropic expansion through its involvement in cellulose microfibril orientation. The Plant Cell.

[50] Wang H, Guo Y, Lv F, Zhu H, Wu S, Jiang Y, Li F, Zhou B, Guo W, Zhang T (2010). The essential role of *GhPEL* gene, encoding a pectate lyase, in cell wall loosening by depolymerization of the de-esterified pectin during fiber elongation in cotton. Plant Mol Biol.

[51] Huang GQ, Gong SY, Xu WL, Li W, Li P, Zhang CJ, Li DD, Zheng Y, Li FG, Li XB (2013). A fasciclin-like arabinogalactan protein, *GhFLA1*, is involved in fiber initiation and elongation of cotton. Plant Physiol.

[52] Harmer SE, Orford SJ, Timmis JN (2002). Characterisation of six alpha-expansin genes in *Gossypium hirsutum* (upland cotton). Mol Gen Genet.

[53] Zhao P-M, Wang L-L, Han L-B, Wang J, Yao Y, Wang H-Y, Du X-M, Luo Y-M, Xia G-X (2009). Proteomic identification of differentially expressed proteins in the Ligon lintless mutant of upland cotton (*Gossypium hirsutum* L.). J Proteome Res.

[54] Kende H, Bradford K, Brummell D, Cho HT, Cosgrove D, Fleming A, Gehring C, Lee Y, McQueen-Mason S, Rose J, Voesenek LA (2004). Nomenclature for members of the expansin superfamily of genes and proteins. Plant Mol Biol.

[55] Marga F, Grandbois M, Cosgrove DJ, Baskin TI (2005). Cell wall extension results in the coordinate separation of parallel microfibrils: evidence from scanning electron microscopy and atomic force microscopy. Plant J.

[56] Marsolais F, Sebastia CH, Rousseau A, Varin L (2004). Molecular and biochemical characterization of BNST4, an ethanol-inducible steroid sulfotransferase from Brassica napus, and regulation of BNST genes by chemical stress and during development. Plant Sci.

[57] Rouleau M, Marsolais F, Richard M, Nicolle L, Voigt B, Adam G, Varin L (1999). Inactivation of brassinosteroid biological activity by a salicylate-inducible steroid sulfotransferase from Brassica napus. J Biol Chem.

[58] Varin L, Marsolais F, Richard M, Rouleau M (1997). Sulfation and sulfotransferases 6: Biochemistry and molecular biology of plant sulfotransferases. FASEB J.

[59] Taliercio EW, Boykin D (2007). Analysis of gene expression in cotton fiber initials. BMC plant biol.

[60] Qin YM, Hu CY, Pang Y, Kastaniotis AJ, Hiltunen JK, Zhu YX (2007). Saturated very-long-chain fatty acids promote cotton fiber and *Arabidopsis* cell elongation by activating ethylene biosynthesis. Plant Cell.

[61] Qin YM, Pujol FM, Shi YH, Feng JX, Liu YM, Kastaniotis AJ, Hiltunen JK, Zhu YX (2005). Cloning and functional characterization of two cDNAs encoding NADPH-dependent 3-ketoacyl-CoA reductased from developing cotton fibers. Cell Res.

[62] Troncoso-Ponce MA, Kilaru A, Cao X, Durrett TP, Fan JL, Jensen JK, Thrower NA, Pauly M, Wilkerson C, Ohlrogge JB (2011). Comparative deep transcriptional profiling of four developing oilseeds. Plant J.

[63] Boerjan W, Ralph J, Baucher M (2003). Lignin biosynthesis. Annu Rev Plant Biol.

[64] Al-Ghazi Y, Bourot S, Arioli T, Dennis ES, Llewellyn DJ (2009). Transcript profiling during fiber development identifies pathways in secondary metabolism and cell wall structure that may contribute to cotton fiber quality. Plant Cell Physiol.

[65] He XC, Qin YM, Xu Y, Hu CY, Zhu YX (2008). Molecular cloning, expression profiling, and yeast complementation of 19 beta-tubulin cDNAs from developing cotton ovules. J Exp Bot.

[66] Loguerico LL, Zhang JQ, Wilkins TA (1999). Differential regulation of six novel MYB-domain genes defines two distinct expression patterns in allotetraploid cotton (*Gossypium hirsutum* L.). Mol Gen Genet.

[67] Pu L, Li Q, Fan XP, Yang WC, Xue YB (2008). The R2R3 MYB Transcription Factor *GhMYB109* Is Required for Cotton Fiber Development. Genetics.

[68] Suo J, Liang X, Pu L, Zhang Y, Xue Y (2003). Identification of *GhMYB109* encoding a R2R3 MYB transcription factor that expressed specifically in fiber initials and elongating fibers of cotton (*Gossypium hirsutum* L.). Biochim Biophys Acta.

[69] Parre E, Geitmann A (2005). More than a leak sealant. The mechanical properties of callose in pollen tubes. Plant Physiol.

