# Supplementary figures and images for: Cotton fiber elongation network revealed by expression profiling of longer fiber lines introgressed with different Gossypium barbadense chromosome segments
Source: BMC Genomics. 2014 Oct 2;15(1):838. doi: 10.1186/1471-2164-15-838 (PMC4190578; doi:10.1186/1471-2164-15-838)

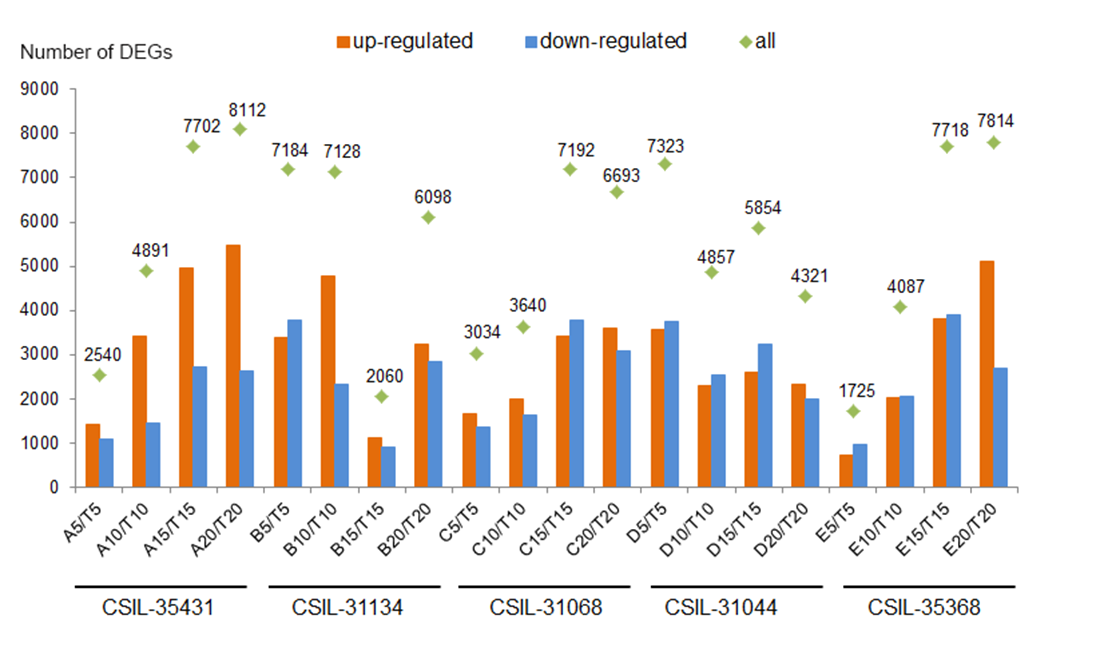

Supplement: Supplementary file 2 — Additional file 2: Figure S1: Statistics of DEGs between CSILs and TM-1 from 5 DPA to 20 DPA. Orange bar, upregulated genes compared to TM-1; blue bar, downregulated genes compared to TM-1, green square, total DEGs. CSILs included CSIL-35431, CSIL-31134, CSIL-31068, CSIL-31044 and CSIL-35368, compared with TM-1. 5, 5 DPA; 10, 10 DPA; 15, 15 DPA; 20, 20 DPA. (TIFF 248 KB) [file 12864_2014_6506_MOESM2_ESM.tiff]

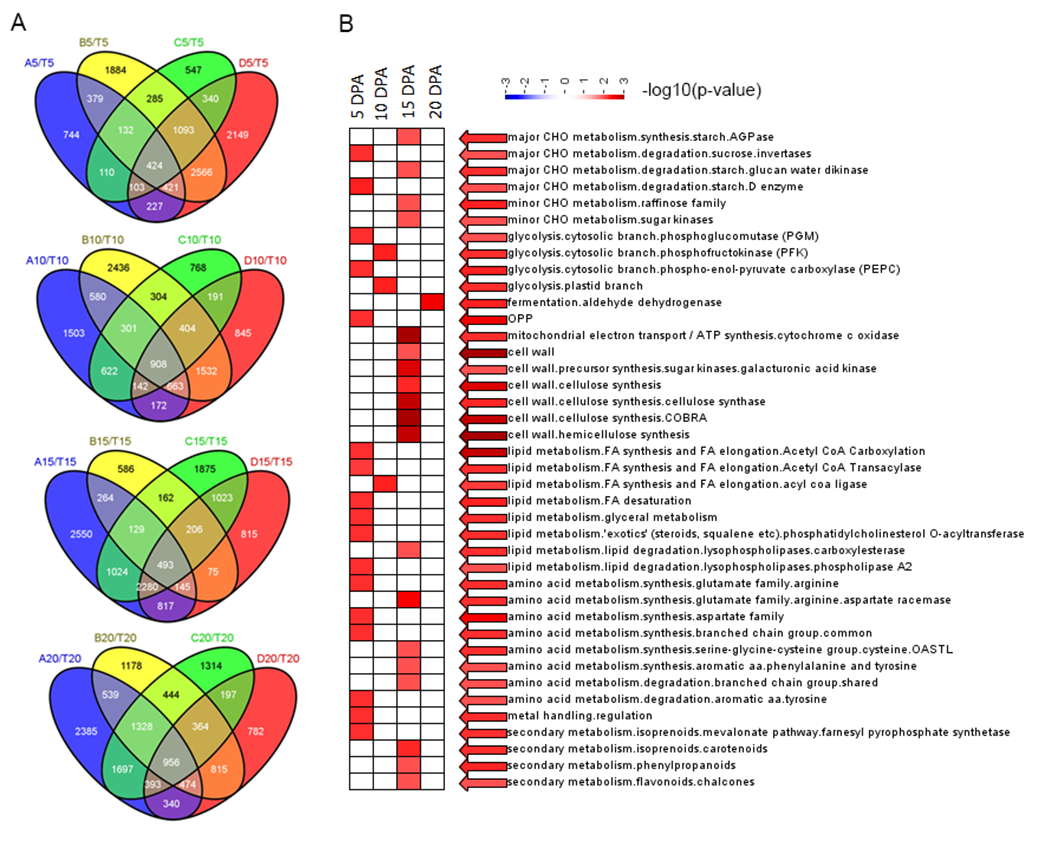

Supplement: Supplementary file 3 — Additional file 3: Figure S2: Functional enrichment analysis of common DEGs between four CSILs with superior fiber quality. (A) Common DEGs between four CSILs from 5 DPA to 20 DPA. A, B, C, D indicated CSIL-35431, CSIL-31134, CSIL-31068, CSIL-31044, respectively. (B) Gene enrichment analysis of common DEGs. Color from blue to red means function enriched with smaller p-value. (TIFF 491 KB) [file 12864_2014_6506_MOESM3_ESM.tiff]

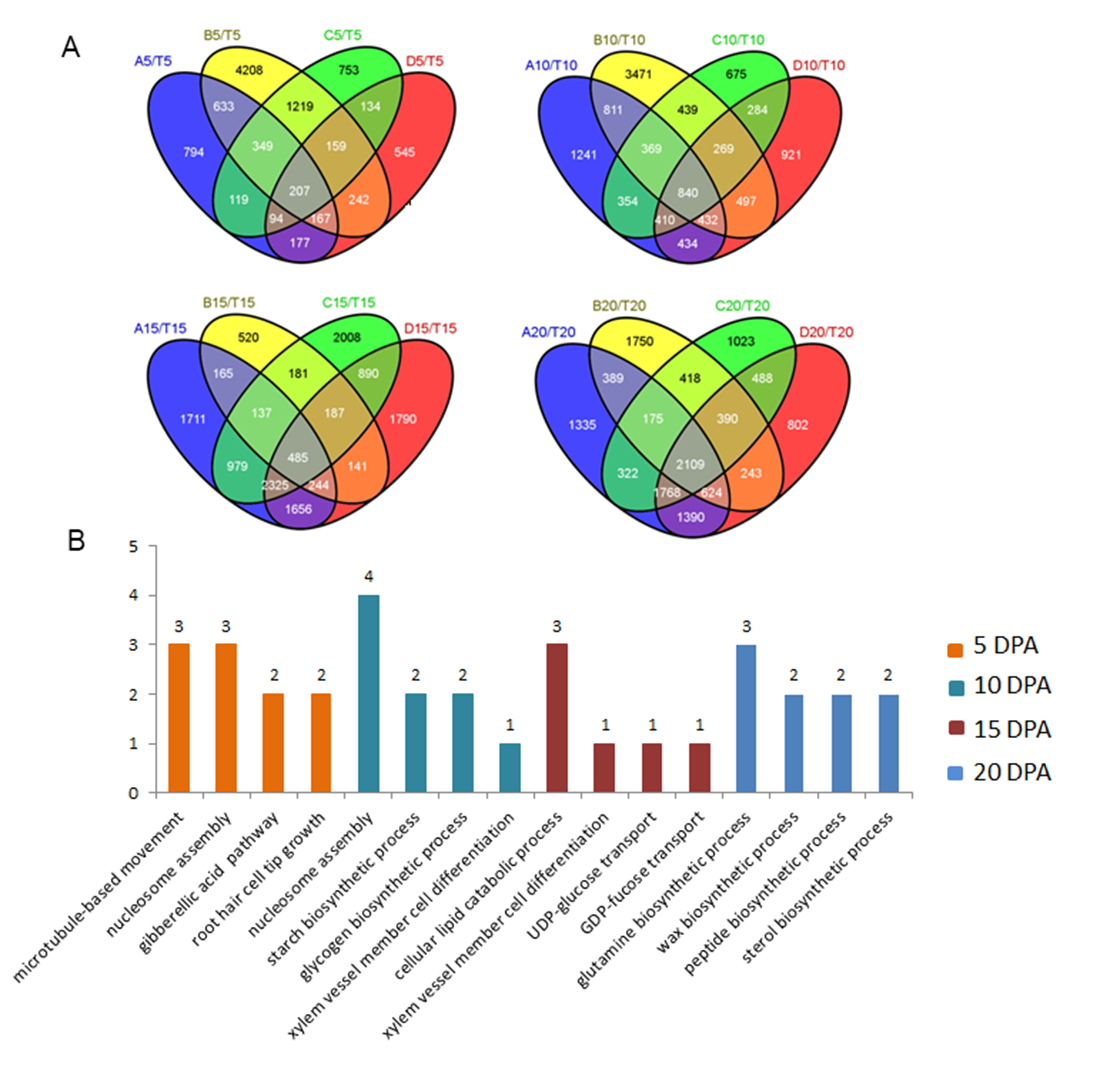

Supplement: Supplementary file 5 — Additional file 5: Figure S3: GO enrichment analysis of genes downregulated only in CSIL-35368. (A) Common DEGs of four CSILs. A, B, C, D indicated CSIL-35431, CSIL-31134, CSIL-31068, CSIL-35368, respectively. 5, 5DPA; 10, 10 DPA; 15, 15 DPA; 20, 20 DPA. (B) GO enrichment of DEGs only down-regulated in CSIL-35368 from 5 to 20 DPA. (TIFF 464 KB) [file 12864_2014_6506_MOESM5_ESM.tiff]

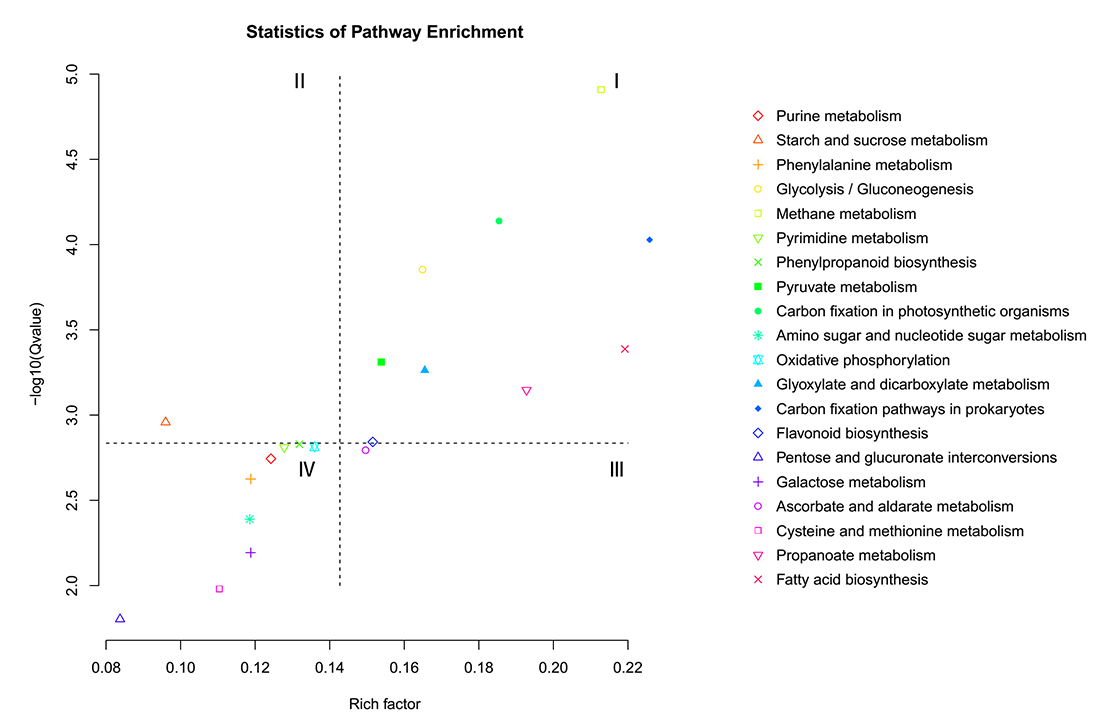

Supplement: Supplementary file 7 — Additional file 7: Figure S4: Pathway analysis of common DEGs between four CSILs from 5 to 20 DPA. All the DEGs used to analysis pathway were according to Figure 2. Twenty pathways were selected by Q-value (corrected P-value). Rich factor means the sample number/reference participated in the same pathway. Significance wasI > II > III > IV. (TIFF 130 KB) [file 12864_2014_6506_MOESM7_ESM.tiff]

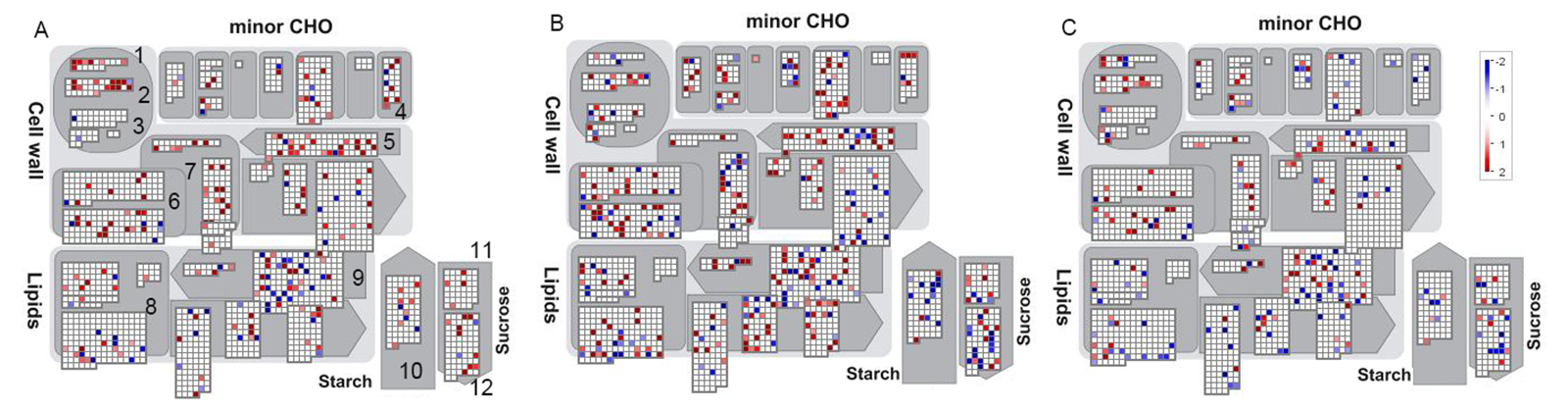

Supplement: Supplementary file 10 — Additional file 10: Figure S5: Overview of metabolism-related DEGs in different CSILs at 10 DPA. (A) Overview of metabolism-related DEGs in CSIL-35431. (B) Overview of metabolism-related DEGs in CSIL-31134. (C) Overview of metabolism-related DEGs in CSIL-35368.1, cell wall proteins, HRGP; 2, cell wall proteins, AGPs; 3, cell wall proteins, LRR; 4, minor CHO metabolism, callose; 5, cell wall precursor synthesis; 6, cell wall pectin esterases and PME; 7, cellulose synthesis; 9, FA synthesis and FA elongation. 10, starch synthesis; 11, starch degradation; 12, sucrose degradation. Blue square, downregulated gene; Red square, upregulated gene. (TIFF 970 KB) [file 12864_2014_6506_MOESM10_ESM.tiff]

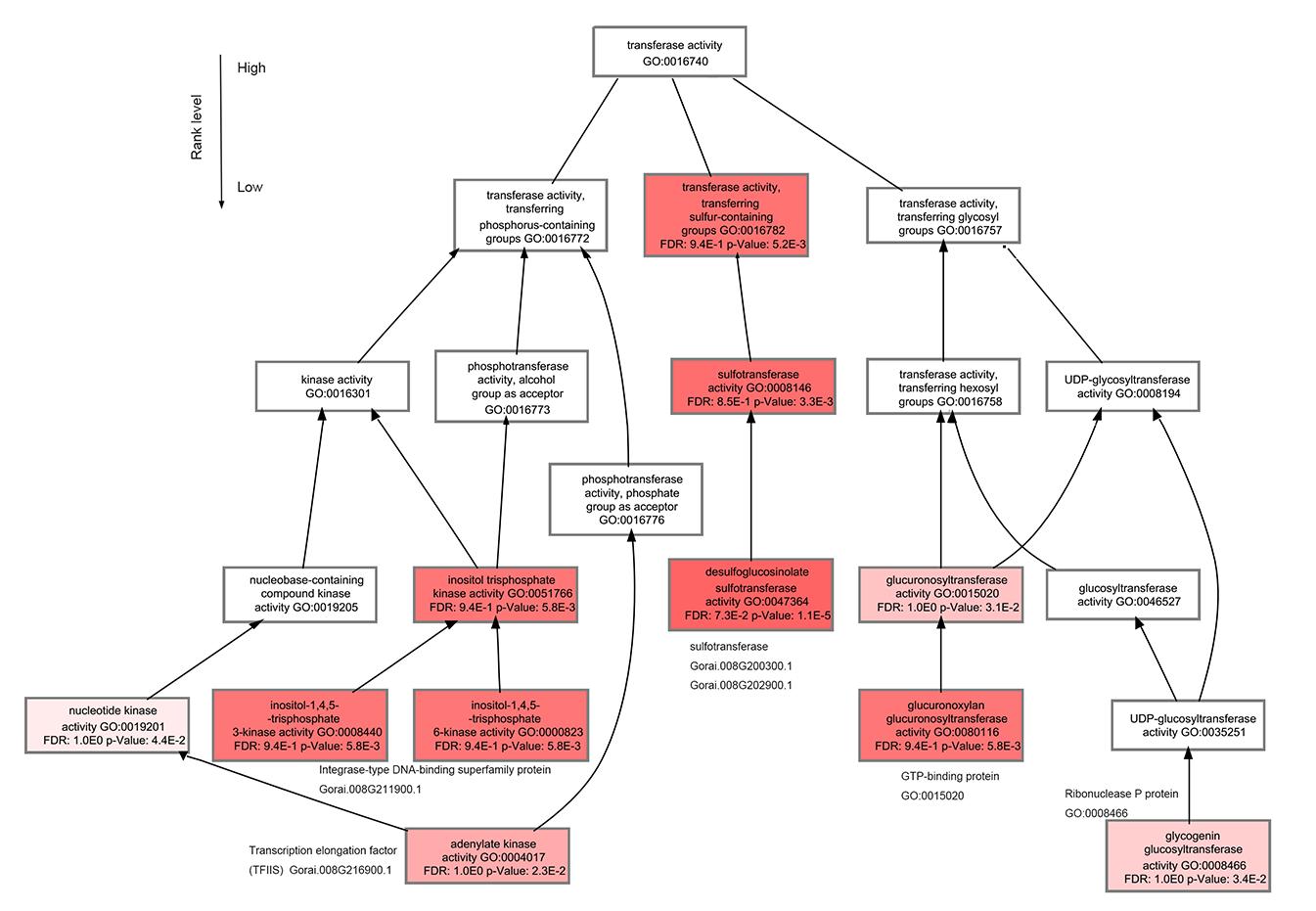

Supplement: Supplementary file 13 — Additional file 13: Figure S6: GO term graph of upregulated DEGs located in introgressed G. barbadense chromosome segments. Every square indicated one molecular function with GO term number. From up to down, the rank level of GO term became lower. With the color deepened, from white to red, the molecular functions were enriched more significantly with smaller P-value. DEGs involved the GO term were listed. (TIFF 305 KB) [file 12864_2014_6506_MOESM13_ESM.tiff]
